# Human cytomegalovirus DNA and immediate early protein 1/2 are highly associated with glioma and prognosis

**DOI:** 10.1007/s13238-020-00696-9

**Published:** 2020-03-18

**Authors:** Le Wen, Fei Zhao, Yong Qiu, Shuang Cheng, Jin-Yan Sun, Wei Fang, Simon Rayner, Michael A. McVoy, Xing-Jun Jiang, Qiyi Tang, Fang-Cheng Li, Fei Hu, Min-Hua Luo

**Affiliations:** 1 Joint Center of Translational Precision Medicine, Guangzhou Institute of Pediatrics, Guangzhou Women and Children’s Medical Center, Guangzhou, 510623 China; 2 State Key Laboratory of Virology, CAS Center for Excellence in Brain Science and Intelligence Technology, Wuhan Institute of Virology, Wuhan, 430071 China; 3 Chinese Institute for Brain Research, Beijing, 102206 China; 4 Wuhan Brain Hospital, Ministry of Transportation, Wuhan, 430010 China; 5 Department of Medical Genetics, Oslo University Hospital and University of Oslo, Oslo, Norway; 6 Department of Pediatrics, Virginia Commonwealth University School of Medicine, Richmond, Virginia USA; 7 Department of Neurosurgery, Xiangya Hospital, Central South University, Changha, 410008 China; 8 Department of Microbiology, Howard University College of Medicine, Washington, DC USA; 9 University of Chinese Academy of Sciences, Beijing, 100049 China

**Dear Editor,**

Gliomas are the most common brain tumors in adults which encompass all primary central nervous system (CNS) tumors of glial cell origin. The World Health Organization (WHO) classifies gliomas into four grades based on the histologic/prognostic features. Because of the unclear etiology and pathogenesis, therapeutic efficacy and prognosis is poor.

Viruses have been identified as causative factors in tumorigenesis. Among nine human herpesviruses (HHVs), Epstein-Barr virus (EBV) and Kaposi’s sarcoma-associated herpesvirus (KSHV) are involved in the development of various cancers. Human cytomegalovirus (HCMV) components have been found to be present in a large proportion of glioblastoma (GBM) (Cobbs et al., 2002). HCMV establishes latency in T98G glioblastoma cells and latent HCMV can be reactivated (Cheng et al., 2017). It was also reported that HCMV potentially induces a functional mesenchymal-to-epithelial (MET) transition without affecting their viability in transformed breast carcinoma and glioma stem cells, which might encourage tumor colonization (Oberstein and Shenk 2017). HCMV has also be shown to be capable of activating oncogenic pathways in mammary epithelial cells (Kumar et al., 2018) and murine cytomegalovirus could promote murine GBM growth via pericyte recruitment and angiogenesis (Krenzlin et al., 2019). Besides a direct role, HCMV inhibits apoptosis and immediate early 1 protein (IE1) increases the malignancy of glioma cells through mediating mitogenicity and converting glioblastoma cells to a stemness phenotype. HCMV proteins US28, pp71, and glycoprotein B (gB) are also involved in gliomagenesis (Dziurzynski et al., 2012). Notably, the administration of valganciclovir, an antiviral used to treat HCMV infection, as an add-on to standard therapy resulted in a higher survival rate among GBM patients in a clinical trial (Soderberg-Naucler et al., 2013). Despite these findings, different viewpoints exist: several researchers consider HCMV components to be absent in GBM (Yamashita et al., 2014) and presented CMV DNA in GBM may be attributed to low-level contamination from adjacent leukocytes (Tang et al., 2015). Other studies consider that HCMV proteins and nucleic acids are present in gliomas, but not correlated with the tumor grade and prognosis (Ding et al., 2014). HCMV viral load and protein levels in glioma are extremely low and variable, which may lead to these incongruous and controversial results. The goal of the present study is to evaluate the correlation between HHVs and glioma in China, where the HCMV seroprevalence is over 90%.

Samples from 378 patients (See Supplementary Materials) were investigated. DNAs of the nine HHVs were examined in paired peripheral whole blood and brain tissues. In peripheral blood HCMV DNA had the highest prevalence, however, no differences in DNAemia between high-grade glioma (HGG), low-grade glioma (LGG), or non-glioma (NG) groups were found for any of the nine HHVs tested (Fig. S1A). In brain tissues the prevalence of viral DNA was generally lower compared with that in peripheral blood. However, a significantly higher prevalence of HCMV DNA was found in HGG vs. LGG and LGG vs. NG groups (Fig. 1A). No difference in HCMV immunoglobulin G (IgG) seroprevalence was found between the three groups (Fig. S1B). These results show that only HCMV was correlated with glioma and this correlation was observed only in brain tissues but not in peripheral blood. One reason for this may be that HCMV reactivation and/or replication could occur primarily locally within the tumor in the context of tumor- or treatment-related immunosuppression. All the patients, whose serum was available, were CMV IgM negative, which suggests that the replication was below the level that is required for triggering an IgM response. HCMV genomic DNA load ranged from 0 to 200 copies/mg tissue in the brain samples which had been identified to be HCMV DNA positive by nested PCR. Significantly higher numbers of HCMV DNA copies were found in HGG tissues compared to LGG or NG tissues (Fig. 1B).

**Figure 1:**
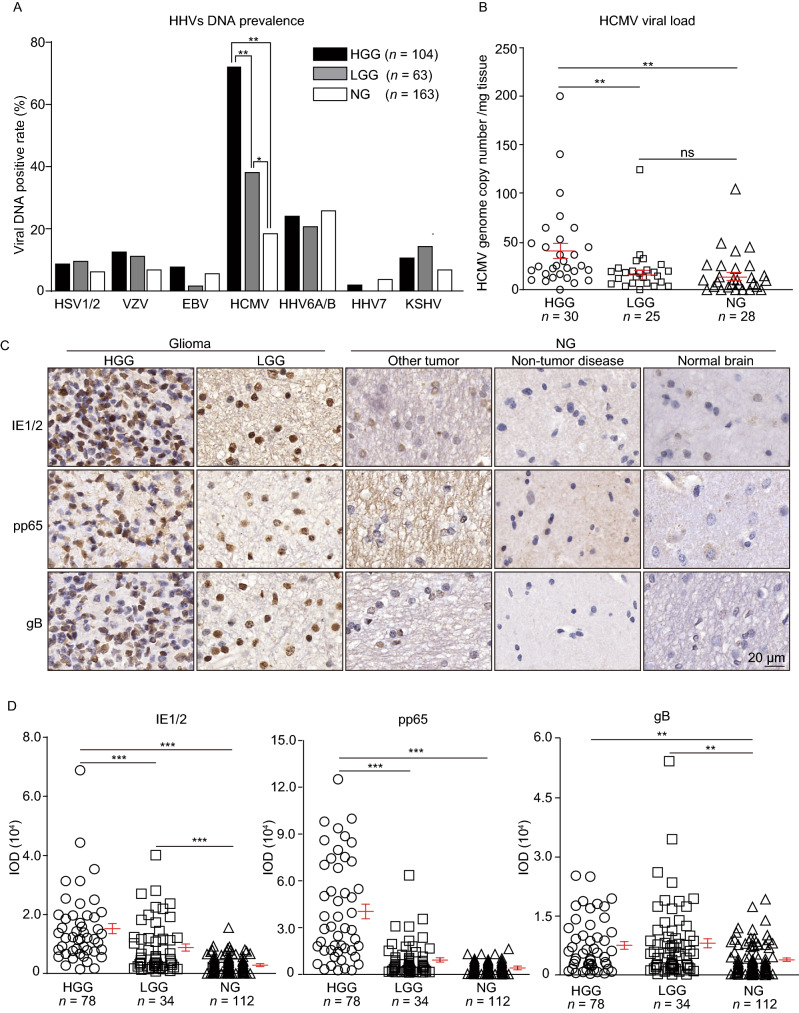

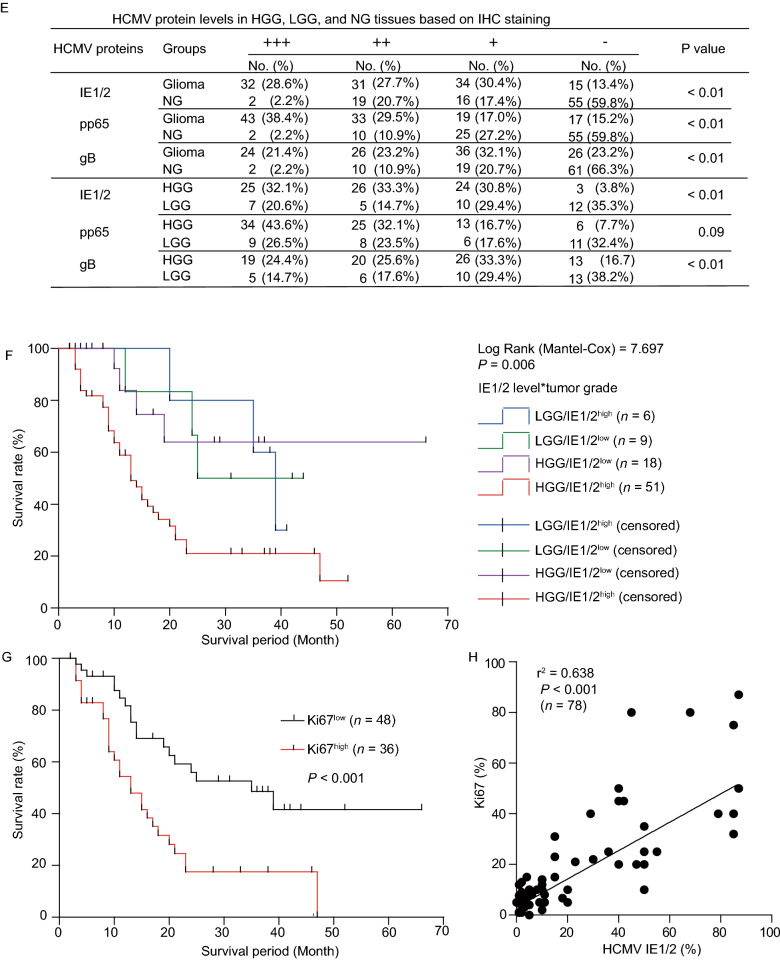
**Association between HHVs, especially HCMV and glioma**. (A) Brain tissue samples from 330 subjects and paired peripheral blood from 145 subjects with high-grade glioma (HGG), low-grade glioma (LGG), or non-glioma (NG) were analyzed by nested PCR to detect the presence of HHV DNA. (B) HCMV genome copy numbers in brain tissues were determined by digital droplet PCR (ddPCR). Horizontal lines show mean ± SEM across all samples and Kruskal-Wallis test with Bonferroni correction was used for comparisons between two groups. (C) Representative images of HCMV proteins IE1/2, pp65, and gB in brain tissues. (D) HCMV protein levels based on IOD analyses of five random fields per specimen after normalizing to the staining background. (E) HCMV protein levels in HGG, LGG, and NG tissues based on IHC scoring system. Significant differences were analyzed by Chi-square test. (F) Analysis of IE1/2 levels and glioma patient survival using Kaplan-Meier curves. Statistical significance was determined using the Log-rank (Mantel-Cox) test. (G) Kaplan-Meier analysis of survival whenglioma patients categorized as having high (+++/++) or low (+/-) levels of Ki67 protein. (H) Correlation between IE1/2 and Ki67 levels in 78 HGGs analyzed by linear regression

As an onco-modulator, HCMV proteins have been reported to promote glioma progression through dysregulating cellular pathways in mutagenesis, apoptosis, angiogenesis, cell invasion, cell stemness, and host antitumor responses. Among them, IE1/2, pp65 (phosphoprotein 65), and gB, representing immediate early, early, and late proteins during infection, were examined in brain tissues by immunohistochemistry (IHC) (Fig. 1C). In NG, only a small fraction of cells were positive for HCMV proteins and the signal was also weaker. In contrast, glioma tissues contained more positive cells and higher signal intensity, suggesting that positive cells in gliomas are more frequent and contain higher HCMV protein levels compared to NG. Quantification of HCMV protein levels based on integral optical density (IOD) and IHC scoring (See Supplementary Materials) confirmed that the levels of IE1/2, pp65 and gB were higher in glioma tissues compared to NG, while IE1/2 levels were higher in both HGG vs. LGG and LGG vs. NG (Fig. 1D and 1E). Odds ratio (OR) estimates obtained from logistic regression revealed only HCMV proteins, including IE1/2, pp65, and gB expression, but not age, sex, or tumor location, are potential risk factors associated with tumor grade (Table S1). Previous studies have mainly focused on HCMV and malignant glioma, especially GBM. The present study conducted a cross-sectional survey of the presence of IE1/2, pp65, and gB in different grades of gliomas and NG. A positive correlation between HCMV genomic DNA and protein levels and glioma implies that HCMV may infect glioma cells and play a role in tumor progression.

The correlation between HCMV and glioma patient survival is somewhat controversial. Ding et al. suggested that HCMV components do not correlate with progression-free survival (Ding et al., 2014), while other studies indicated that HCMV IE protein expression is inversely correlated with GBM patient survival (Fornara et al., 2016). The relationships were analyzed between the HCMV protein levels and post-surgical survival of 84 glioma patients whose survival information was available. Tumors from these 84 patients were categorized according to the IE1/2 levels, and then assigned to four groups based on tumor grade and IE1/2 level: LGG/IE1/2^low^, LGG/IE1/2^high^, HGG/IE1/2^low^, and HGG/IE1/2^high^ (Table S2). Cox regression was performed to analyze correlative factors, including age, sex, and tumor grade, and protein levels of IE1/2, pp65, and gB. Significant association for IE1/2 levels with tumor grade and prognosis was confirmed, but not for all other factors (Table S3). A significant association remained between IE1/2 levels, tumor grade, and the survival when other factors were removed. There was also a significant difference between the HGG/IE1/2^high^ and the other three groups (Table S4), indicating that HGG patients with high IE1/2 levels had significantly shorter survival compared to the other groups. However, no significant differences were observed between LGG patients with IE1/2^low^ or IE1/2^high^ tumors (Fig. 1F). These results indicate that IE1/2 expression and tumor grade mutually reinforce each other in HGG and IE1/2 might have onco-modulatory effects that vary with tumor grade. This is a complicated process that occurs within the host and why this association does not extend to pp65 or gB remains unclear. While the data suggest that patients with LGG/IE1/2^high^ tumors may have better 19-month survival than those with HGG/IE1/2^high^ (Table S2), the small size of the LGG/IE1/2^high^ group and limited follow-up period make it uncertain whether their long-term survival is different from the HGG/IE1/2^high^ group. Expression of Ki67 was examined as a measure of proliferation. Higher Ki67 levels were observed in HGGs (Table S5) and also, higher Ki67 levels correlated with shorter survival (Fig. 1G). Ki67 and IE1/2 were significant prognostic markers for glioma patients. Linear regression analysis showed a positive correlation between IE1/2 and Ki67 expression (Fig. 1H). Whether Ki67 and IE1/2 are interdependent needs to be specified.

To investigate the distribution of IE1/2-positive cells in gliomas, surgically resected samples from cases of GBM were identified as tumor, peritumoral, or areas of adjacent normal-appearing brain. In the tumor tissue necrotic areas were observed, as well areas with abnormal cell proliferation and vascular proliferation or angiogenesis (Fig. 2A). Examination at higher magnification revealed that cell proliferation and angiogenesis declined with increasing distance from the tumor and thus were progressively less frequent in peritumoral areas and adjacent normal-appearing brain (Fig. 2A–C). Also, higher Ki67 levels were found in tumor regions compared to peritumoral regions, while the Ki67 signal was barely detectable in areas of adjacent normal-appearing brain far distal from the tumoral areas (Fig. 2D). Similarly, IE1/2-positive cells were primarily located in tumoral regions and both the prevalence of IE1/2-expressing cells and their levels of IE1/2 declined with distance from the tumoral regions (Fig. 2E). Notably, IE1/2-expressing cells were concentrated in parenchymal tissues while relatively few were associated with blood vessels (Fig. 2F). This tumor-preferential distribution of IE1/2 has been reported in other studies. IE1/2 staining in primary HGG cells and tissues was observed in the cytoplasm as well as in the nucleus (Fig. S1C and S1D), which contrasts with the exclusively nuclear localization of IE1/2 in infected fibroblasts (Fig. S1E). This phenomenon has also been described by others(Cobbs et al., 2002). One possible explanation is that in some glioma cells differential splicing of the IE gene locus could result in expression of IE protein isoforms that localize to the cytoplasm while retaining the epitope that is recognized by the IE1/2-specific antibody. Indeed, minor IE isoforms, including IE38, IE55, and IE18, have been observed during fibroblast infection and a similar isoform may be involved with genome maintenance during latency (Tarrant-Elorza et al., 2014). This hypothesis, and its physiological relevance, requires further investigation.

**Figure 2:**
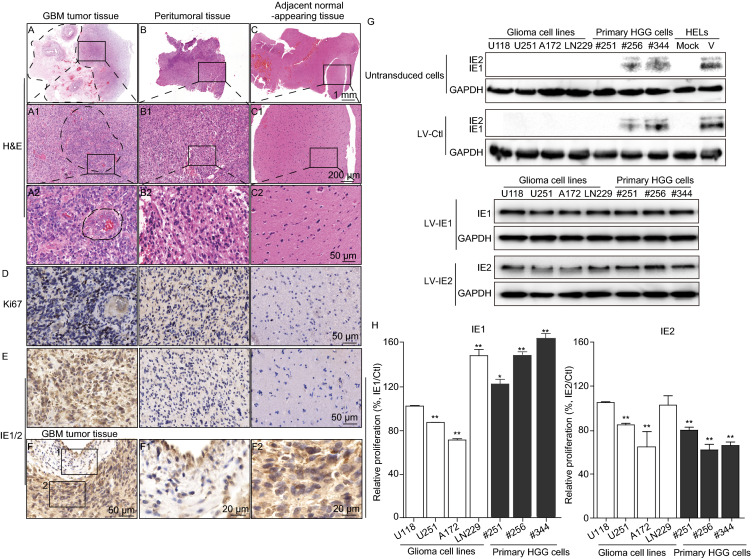

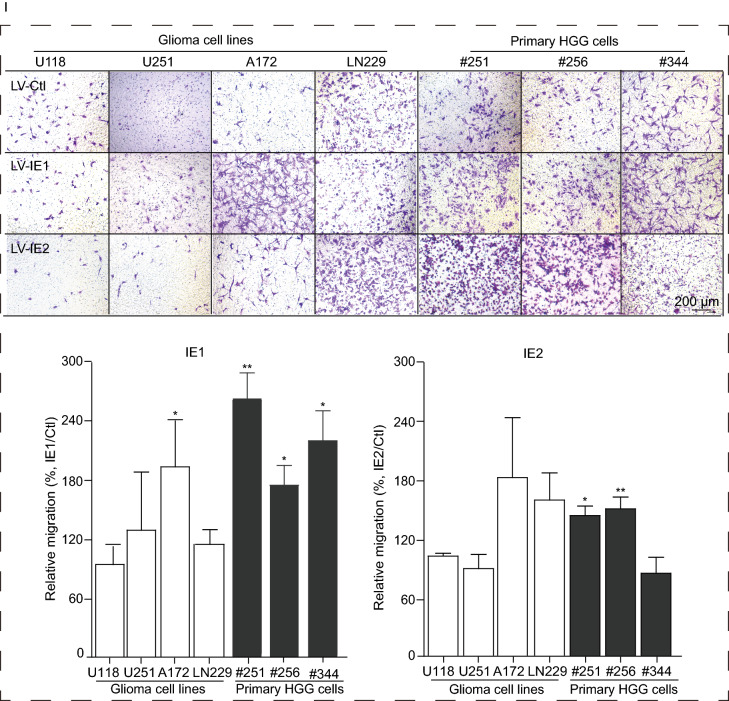
**IE1/2 distribution in GBM and its impact on glioma cell proliferation and migration**. Regions within brain tissue resected from a representative GBM patient were identified as tumoral (A), peritumoral (B), or areas of adjacent normal-appearing brain (C) based on histopathology of H&E stained sections. In the tumor tissue, a necrotic area is enclosed by dashed line and an area of abnormal cell proliferation is indicated by a rectangle (A). In A1 the area of abnormal cell proliferation is enlarged to show an area of vascular proliferation or angiogenesis enclosed by a dashed line. Further magnification in A2 shows vascular proliferation or angiogenesis enclosed by a double-dashed line. For comparison, similar magnifications are shown of regions identified as peritumoral (B1, B2) or adjacent normal-appearing brain (C1-C2). Ki67 (D) and IE1/2 (E) staining within representative regions. (F) IE1/2 proteins distribution differs between a blood vessel (F1) and parenchyma (F2) in tumor region. (G) Expression levels of IE1 or IE2 in untransduced glioma cells or cells transduced with LV-Ctl, LV-IE1, or LV-IE2; mock- and HCMV-infected (V) HELs worked as negative and positive controls, respectively. HELs infected by HCMV were collected at 12 hpi at a MOI = 0.01. (H) The impact of IE1 or IE2 on cell proliferation. (I) The impact of IE1 or IE2 on cell migration

Given the above described associations between tumor grade, IE1/2 expression, and survival, we investigated the biological effects of IE1 and IE2 on glioma cell growth. Four glioma cell lines (U118, U251, A172, and LN229), as well as primary glioma cells derived from HGG tissues of three different cases (#251, #256, and #344) were evaluated. When cells or the cells transduced with an empty vector lentivirus control (LV-Ctl) were evaluated for IE1/2 expression, cells derived from tumors #256 and #344 contained low levels of IE1 and IE2, whereas the four glioma cell lines and cells from tumor #251 were negative. In contrast, the cells transduced with LV-IE1 or LV-IE2 had much higher levels of IE1 or IE2 and levels were consistent across all IE1- or IE2-transduced cultures (Fig. 2G, note that exposure time is longer for untransduced and LV-Ctl-transduced cells). Data showed that IE1 promoted proliferation of all three primary glioma cells (#251, #251, and #344) and one glioma cell line LN229, decreased proliferation of U251 and A172, and did not affect U118 (Fig. 2H). In contrast, IE2 decreased proliferation of all three primary glioma cells and two glioma cell lines but had no effect on the other two (Fig. 2H). In addition, differential effects of IE1 on different GBM cell lines has also been reported (Cobbs et al., 2008). This differential response of GBM cell lines to IE1 might result from prevalent genetic lesions in signaling and cell cycle regulatory proteins, which could influence induction of cytoplasmic mitogenic signaling pathways through regulation of AKT and MAPK activity (Cobbs et al., 2008). Correlation between Ki67 and IE1/2 protein levels also suggests that IE1 may play a causative role in promoting proliferation of HGG cells *in vivo*. IE proteins lead to a transcriptional cascade necessary for the production of infectious HCMV and participate in many cellular or viral regulatory pathways as transcription factors. It has also been shown that EBV latency-associated proteins may drive a minority of infected cells to develop into tumors (Young et al., 2016). Consistent with the tumorigenic effects of EBV proteins, HCMV IE1 is also involved in its latency in hematopoietic progenitor cells (Tarrant-Elorza et al., 2014) and promotes cell cycle entry and DNA synthesis of human glioma cell lines (Dziurzynski et al., 2012). Migration was also measured and representative images were shown (Fig. 2I). IE1 enhanced migration of all three primary glioma cells and one glioma cell line (A172), but did not significantly alter migration of the other three glioma cell lines. Similarly, IE2 enhanced the migration of two primary glioma cells (#251 and #256) and two glioma cell lines (A172 and LN229), but had no significant effect on the third primary glioma cells or the other two glioma cell lines (Fig. 2I). IE1 and IE2 are also reported to promote degradation of connexin-43 and disruption of gap junction communication in U373MG (Dziurzynski et al., 2012) and inhibit apoptosis and to cooperate with E1A to sponsor “hit-and-run” transformation. Our data suggest that IE1 more than IE2 may contribute to glioma malignant progression and may be involved in clinical outcome, as suggested by the survival analysis. HCMV IE1 protein is considered to be a key viral antagonist of intrinsic immune responses of host cells, which counteracts antiviral restriction via STAT binding and PML-NBs targeting in order to antagonize the IFN- mediating signaling. It has also been reported that tumor suppressor promyelocytic leukemia (PML) expression is decreased in multiple human cancers, including glioma (Gurrieri et al., 2004). Thus, it is possible that the high expression of IE1 in glioma tissue may participate in creating an immunosuppressed state through inhibiting innate immune responses of the host, which leads to a favorable environment for immune-evasion and cell growth of glioma.

The present work extends the body of evidence linking HCMV and glioma, and further suggests that IE1 may promote progression and may provide a useful prognostic marker. As clinical benefit has been reported for ganciclovir or valganciclovir in treatment of GBM patients (Soderberg-Naucler et al., 2013), it may be helpful to stratify glioma patients based on IE1 or IE2 levels in their tumors and then administer different treatments accordingly.

## References

[CR1] Cheng S, Jiang X, Yang B, Wen L, Zhao F, Zeng WB, Liu XJ, Dong X, Sun JY, Ming YZ (2017). Infected T98G glioblastoma cells support human cytomegalovirus reactivation from latency. Virology.

[CR2] Cobbs CS, Harkins L, Samanta M, Gillespie GY, Bharara S, King PH, Nabors LB, Cobbs CG, Britt WJ (2002). Human cytomegalovirus infection and expression in human malignant glioma. Cancer Res.

[CR3] Cobbs CS, Soroceanu L, Denham S, Zhang WY, Kraus MH (2008). Modulation of oncogenic phenotype in human glioma cells by cytomegalovirus IE1-mediated mitogenicity. Cancer Res.

[CR4] Ding DL, Han S, Wang ZX, Guo ZZ, Wu AH (2014). Does the existence of HCMV components predict poor prognosis in glioma?. J Neuro-Oncol.

[CR5] Dziurzynski K, Chang SM, Heimberger AB, Kalejta RF, McGregor Dallas SR, Smit M, Soroceanu L, Cobbs CS, HCMV and Gliomas Symposium (2012). Consensus on the role of human cytomegalovirus in glioblastoma. Neuro Oncol.

[CR6] Fornara O, Bartek J, Rahbar A, Odeberg J, Khan Z, Peredo I, Hamerlik P, Bartek J, Stragliotto G, Landazuri N (2016). Cytomegalovirus infection induces a stem cell phenotype in human primary glioblastoma cells: prognostic significance and biological impact. Cell Death Differ.

[CR7] Gurrieri C, Capodieci P, Bernardi R, Scaglioni PP, Nafa K, Rush LJ, Verbel DA, Cordon-Cardo C, Pandolfi PP (2004). Loss of the tumor suppressor PML in human cancers of multiple histologic origins. J Natl Cancer Inst.

[CR8] Krenzlin H, Behera P, Lorenz V, Passaro C, Zdioruk M, Nowicki MO, Grauwet K, Zhang H, Skubal M, Ito H (2019). Cytomegalovirus promotes murine glioblastoma growth via pericyte recruitment and angiogenesis. J Clin Invest.

[CR9] Kumar A, Tripathy MK, Pasquereau S, Al Moussawi F, Abbas W, Coquard L, Khan KA, Russo L, Algros MP, Valmary-Degano S (2018). The human cytomegalovirus strain db activates oncogenic pathways in mammary epithelial cells. EBioMedicine.

[CR10] Oberstein A, Shenk T (2017). Cellular responses to human cytomegalovirus infection: Induction of a mesenchymal-to-epithelial transition (MET) phenotype. Proc Natl Acad Sci USA.

[CR11] Soderberg-Naucler C, Rahbar A, Stragliotto G (2013). Survival in patients with glioblastoma receiving valganciclovir. N Engl J Med.

[CR12] Tang KW, Hellstrand K, Larsson E (2015). Absence of cytomegalovirus in high-coverage DNA sequencing of human glioblastoma multiforme. Int J Cancer.

[CR13] Tarrant-Elorza M, Rossetto CC, Pari GS (2014). Maintenance and replication of the human cytomegalovirus genome during latency. Cell Host Microbe.

[CR14] Yamashita Y, Ito Y, Isomura H, Takemura N, Okamoto A, Motomura K, Tsujiuchi T, Natsume A, Wakabayashi T, Toyokuni S (2014). Lack of presence of the human cytomegalovirus in human glioblastoma. Mod Pathol.

[CR15] Young LS, Yap LF, Murray PG (2016). Epstein-Barr virus: more than 50 years old and still providing surprises. Nat Rev Cancer.

