# Correction to: Human cytomegalovirus DNA and immediate early protein 1/2 are highly associated with glioma and prognosis

**DOI:** 10.1007/s13238-020-00787-7

**Published:** 2020-09-15

**Authors:** Le Wen, Fei Zhao, Yong Qiu, Shuang Cheng, Jin-Yan Sun, Wei Fang, Simon Rayner, Michael A. McVoy, Xing-Jun Jiang, Qiyi Tang, Fang-Cheng Li, Fei Hu, Min-Hua Luo

**Affiliations:** 1 Joint Center of Translational Precision Medicine, Guangzhou Institute of Pediatrics, Guangzhou Women and Children’s Medical Center, Guangzhou, 510623 China; 2 State Key Laboratory of Virology, CAS Center for Excellence in Brain Science and Intelligence Technology, Wuhan Institute of Virology, Wuhan, 430071 China; 3 Chinese Institute for Brain Research, Beijing, 102206 China; 4 Wuhan Brain Hospital, Ministry of Transportation, Wuhan, 430010 China; 5 Department of Medical Genetics, Oslo University Hospital and University of Oslo, Oslo, Norway; 6 Department of Pediatrics, Virginia Commonwealth University School of Medicine, Richmond, Virginia USA; 7 Department of Neurosurgery, Xiangya Hospital, Central South University, Changha, 410008 China; 8 Department of Microbiology, Howard University College of Medicine, Washington, DC USA; 9 University of Chinese Academy of Sciences, Beijing, 100049 China

## Correction to: Protein Cell 2020, 11(7):525–533 10.1007/s13238-020-00696-9

In the original publication the email addresses of corresponding authors have not been displayed. The correct email addresses of corresponding authors are provided in this correction. Fang-Cheng Li (sjwklfc@126.com), Fei Hu (neuron111@163.com), Min-Hua Luo (luomh@wh.iov.cn).

